# An IgM monoclonal antibody targeting diguanylate cyclase DgcE potentiates gentamicin activity against avian pathogenic *Escherichia coli* through modulation of c-di-GMP signaling

**DOI:** 10.1186/s12917-026-05548-y

**Published:** 2026-06-01

**Authors:** Li Fangfang, Nie Lianhua, Wang haiyang, Wang Zhihao, Lian Liyan, Hu Jiangang, Jia yuanzheng, Bao Yinli, Han xiangan, Wang haidong

**Affiliations:** 1 College of Veterinary Medicine, Shanxi Agricultural University, Taigu, 030801 P. R. China; 2 Shanghai Veterinary Research Institute, The Chinese Academy of Agricultural Sciences (CAAS), 518 Ziyue Road, Shanghai, 200241 China; 3 Key Laboratory of Fujian Universities Preventive Veterinary Medicine and Biotechnology, Longyan University, Longyan, 364012 China

**Keywords:** Avian Pathogenic *Escherichia coli* (APEC), Diguanylate cyclase DgcE, Monoclonal antibody, Aminoglycoside resistance, Cyclic di-GMP

## Abstract

**Background:**

The combination of monoclonal antibodies with aminoglycosides represents a promising strategy to counter the increasing prevalence of aminoglycoside resistance in avian pathogenic *E. coli* (APEC). Although the diguanylate cyclase DgcE (UniProt: P38097) is known to regulate virulence through c-di-GMP signaling, its potential as a therapeutic target has not been investigated.

**Results:**

In this study, five female BALB/c mice were immunized to generate a monoclonal antibody. We developed an IgM monoclonal antibody (E11G12) targeting a defined region of DgcE and evaluated its ability to potentiate gentamicin activity. Structural prediction and docking analyses suggested specific binding interactions within the targeted DgcE region. In vitro checkerboard assays demonstrated synergistic activity between E11G12 and gentamicin (FICI ≤ 0.5), and time-kill assays showed enhanced bactericidal activity compared to monotherapy. Treatment was associated with reduced intracellular c-di-GMP levels and increased gentamicin accumulation.

**Conclusion:**

These findings suggest that the E11G12-aminoglycoside combination exhibits synergistic anti-APEC activity, potentially through blocking virulence via DgcE inhibition and enhancing antibiotic efficacy in association with c-di-GMP suppression. This approach offers a novel therapeutic strategy against drug-resistant APEC infections.

**Supplementary Information:**

The online version contains supplementary material available at 10.1186/s12917-026-05548-y.

## Introduction

Avian pathogenic *Escherichia coli* (APEC) causes severe respiratory and systemic infections in poultry, resulting in annual economic losses exceeding $3 billion worldwide [1]. Aminoglycosides such as gentamicin are still widely used as first-line treatments; however, resistance rates exceed 40% in clinical isolates, largely due to the widespread dissemination of resistance genes such as *aac(3)-II* and *aph(4)-Ia* [2, 3]. This challenge is further exacerbated by biofilm formation—a major virulence mechanism that enhances antibiotic tolerance by up to 1,000-fold through limited drug penetration, altered metabolic states, and induction of phenotypic resistance [4, 5]. Biofilm development follows a five-stage process: reversible adhesion, irreversible attachment mediated by adhesins, production of an extracellular polymeric substance (EPS) matrix, maturation regulated by quorum sensing, and controlled dispersal [6]. The second messenger cyclic di-GMP (c-di-GMP) is a central regulator of biofilm formation, motility suppression, and envelope-associated phenotypes in Gram-negative bacteria [7, 8].

The transmembrane diguanylate cyclase DgcE (UniProt: P38097), which contains 11 transmembrane domains (TMDs), functions as a central node for c-di-GMP production in APEC. Our previous studies showed that ΔDgcE isogenic mutants display a 48% reduction in biofilm formation and a 3.2-fold increase in motility [9]. Transcriptomic analysis further indicated significant downregulation of the biofilm-associated adhesion gene agn43 (62%) and upregulation of flagellar biosynthesis genes *fliA* and *fliC* (2.8-fold), supporting DgcE’s dual role in promoting biofilm formation and suppressing motility [10]. In addition, mutants exhibited altered RDAR (red, dry, and rough) colony morphology, suggesting impaired EPS matrix assembly [11]. Although DgcE is conserved across 48 clinical APEC strains and plays a critical regulatory role, no therapeutic agents currently target this membrane-embedded synthase. Targeting DgcE may therefore represent a strategy to modulate c-di-GMP-dependent phenotypes that influence antibiotic susceptibility. Existing small-molecule inhibitors of diguanylate cyclases often exhibit off-target effects and poor bioavailability [12], while conventional IgG antibodies are generally unable to engage conformational epitopes within multi-transmembrane protein complexes.

We hypothesize that an anti-DgcE monoclonal antibody (mAb) targeting extracellular domains may suppress c-di-GMP–mediated virulence and restore APEC susceptibility to aminoglycosides. This hypothesis is supported by two proposed mechanisms: (ⅰ) antibody binding to the extracellular TM3–TM4 loop could allosterically inhibit the cyclase activity of DgcE, thereby lowering c-di-GMP levels and derepressing flagellar motility, which in turn impedes biofilm initiation; and (ⅱ) depletion of c-di-GMP may increase membrane permeability, promoting aminoglycoside uptake [13]. In support of this concept, IgM-class antibodies demonstrate enhanced avidity for multimeric membrane proteins owing to their pentameric flexibility [14]. This property has been successfully leveraged in clinical settings, as exemplified by the anti-MRSA IgM tesobart, which restores β-lactam susceptibility through targeting wall teichoic acid [15].

Therefore, this study aimed to develop and characterize the first IgM monoclonal antibody (designated E11G12) targeting an extracellular epitope of DgcE. We performed epitope mapping to identify its binding site, assessed its synergistic effects with aminoglycoside antibiotics using checkerboard assays, and systematically validated the dual mechanism—whereby inhibition of DgcE function combined with enhanced antibiotic uptake restores APEC susceptibility—through c-di-GMP quantification, bacterial uptake assays, and cellular infection models. These investigations were conducted to evaluate the potential of this antibody as a novel adjunctive therapy against drug-resistant APEC infections.

## Materials and methods

### Bacterial strains, cell lines, and culture conditions

The APEC clinical isolate DE17 (serotype O2; NCBI accession: ASM1686417v1) served as the parental strain, while *E. coli* BL21(DE3) (TransGen Biotech, CD601) was used for protein expression. For the bacterial two-hybrid (BACTH) assay, *E. coli* BTH101 (laboratory stock) was utilized. The SP2/0 myeloma cell line (ATCC^®^ CRL-1581™) and RAW264.7 macrophages (laboratory stock) were used for monoclonal antibody production and cell infection assays, respectively. All bacterial strains were cultured Luria-Bertani (LB) broth (10 g/L tryptone, 5 g/L yeast extract, 10 g/L NaCl) or on LB agar at 37 °C with shaking at 180 rpm. For antimicrobial synergy assays, Mueller-Hinton broth (MHB) (Oxoid, UK) was used.

### Bioinformatic analysis of DgcE

Physicochemical properties and functional domains of DgcE (UniProt: P38097) were predicted using ProtParam (https://web.expasy.org/protparam/) for molecular weight/pI analysis, SMART (http://smart.embl-heidelberg.de/) for domain prediction, and TMHMM 2.0 for transmembrane domain (TMD) mapping. Signal peptide prediction was performed using SignalP-5.0. These analyses were conducted to characterize the full-length DgcE protein (1105 amino acids) and to identify its 11 predicted transmembrane regions.

### Cloning, expression, and purification of DgcE

The C-terminal fragment of DgcE (1,280 bp containing GGDEF/EAL domains) was amplified from DE17 genomic DNA using primers (dgcE-F: CGCGGATCCATGTATACGAAGATTATTGGTACTGG and dgcE-R: CCCAAGCTTCTAGAAACGAACCAGCG) with *Bam*HⅠ/*Eco*RⅠ restriction sites (Table 1), purified with Gel Extraction Kit (Qiagen, 28704), and ligated into pColdⅠ vector (Takara, 3361) using T4 DNA Ligase (Thermo Fisher, EL0014) after double digestion (37 °C, 2 h); the construct was transformed into *E. coli* BL21(DE3) and verified by sequencing (Sangon Biotech). For protein expression, a single colony of BL21(DE3)-pColdⅠ-dgcE was cultured in 500 mL of LB broth containing ampicillin (100 µg/mL) at 37 °C until the OD_600_ reached 0.6–0.8. Expression was induced by adding 0.5 mM IPTG (Isopropyl β-D-1-thiogalactopyranoside, Sigma, I6758) and continued at 16 °C for 24 h with shaking at 180 rpm. Cells were harvested by centrifugation (12,000 rpm, 10 min, 4 °C), resuspended in lysis buffer (50 mM Tris-HCl, 300 mM NaCl, pH 8.0), and disrupted by ultrasonication (20 kHz, 5 min, with 5 s on/off cycles) on ice. The lysate was centrifuged (12,000 rpm, 20 min, 4 °C) to separate the supernatant. The recombinant DgcE protein, which carries a His-tag, was purified using Ni-NTA affinity chromatography (GE Healthcare, 17531801). The supernatant was loaded onto a column containing 3 mL of BeyoGold™ His-tag Purification Resin. The column was washed with increasing concentrations of imidazole (from 15 mM to 250 mM) to remove nonspecifically bound proteins, and the target protein was eluted in 100–250 mM imidazole. The eluted fractions containing pure DgcE were pooled and dialyzed against 1× PBS (phosphate-buffered saline) at 4 °C for 48 h to remove imidazole. The purified protein concentration was determined using the BCA Protein Assay Kit (Thermo Fisher, 23225), and its purity was assessed by SDS-PAGE.

### c-di-GMP synthase activity assay

The dual-fluorescence reporter plasmid pRP0122-atr was electroporated into BL21 harboring pColdⅠ-DgcE, induced with 0.5 mM IPTG (Sigma, I6758) at 25 °C for 18 h, fixed with 4% paraformaldehyde for 10 min, and visualized under fluorescence microscopy (Olympus BX53). For quantitative analysis, fluorescence intensities of TurboRFP and AmCyan were measured from at least three independent fields per sample using ImageJ software. For quantitative analysis, fluorescence intensities of TurboRFP and AmCyan were measured from at least three independent fields per sample using ImageJ software. The TurboRFP/AmCyan ratio was calculated to normalize for variations in transformation efficiency and expression. Each experiment was performed in three independent biological replicates (*n* = 3), and statistical significance was assessed using one-way ANOVA with Tukey’s post-hoc test (*P* < 0.05 considered significant).

### Monoclonal antibody production

Monoclonal antibodies (mAbs) against DgcE were generated using the standard hybridoma technology. Five female six-week-old BALB/c mice (Vital River Laboratory) were immunized subcutaneously with 50 µg DgcE emulsified in Freund’s complete adjuvant (Sigma, F5881), followed by two biweekly booster immunizations with 50 µg of antigen emulsified in Freund’s incomplete adjuvant (Sigma, F5506). Mice were housed in groups of five per cage under a 12 h light/dark cycle at 22 ± 2 °C and 50 ± 10% humidity, with free access to food and water. One week after the second boost, serum was collected from the mice via the retro-orbital plexus under isoflurane anesthesia (3% induction, 1.5% maintenance in 100% oxygen). Serum titers were determined by indirect ELISA, using 2 µg/well of DgcE protein as the coating antigen (overnight at 4℃). All serum samples were tested in three technical replicates. The cut-off titer was defined as the highest dilution yielding an OD_450_ value at least 2.1 times that of the negative control. The results are presented as mean ± SD. The mouse with the highest serum titer was selected for a final intraperitoneal boost with 200 µg of antigen (without adjuvant) three days before cell fusion. Splenocytes were harvested from the immunized mouse and fused with SP2/0 myeloma cells (ATCC^®^ CRL-1581™) at a ratio of 5:1 using 50% polyethylene glycol (PEG) 1500 (Roche, 10783641001). Fused cells were selected in HAT medium (Hypoxanthine-Aminopterin-Thymidine, Sigma, H0262) and cultured in 96-well plates on a feeder layer of KM mouse peritoneal macrophages. Hybridoma supernatants were screened for DgcE-specific antibodies by indirect ELISA. Positive clones were subcloned three times by limiting dilution to ensure monoclonality. One stable clone producing an IgM isotype antibody (designated E11G12) was obtained. The monoclonal antibody (E11G12) was purified from culture supernatants using HiTrap IgM columns (Cytiva, 17308021) following the manufacturer’s instructions. The purified antibody was quantified and stored at -80℃ until use. At the end of the experiment, all mice were euthanized by cervical dislocation under deep isoflurane anesthesia.

No randomisation or blinding was applied because all mice received the same immunization protocol and only the mouse with the highest serum titer was selected for fusion. The sample size (*n* = 5) was chosen based on standard hybridoma protocols without a priori power calculation.

### Antibody characterization

Antibody isotype was confirmed with Mouse mAb Isotyping Kit (Sigma, ISO2); epitope mapping utilized peptide arrays (DgcE_(210-280),_ 15-mer/3-aa overlap; GL Biochem) probed with E11G12 (10 µg/mL); variable regions were amplified with degenerate primers and sequenced (MetWare Biotechnology). The deduced amino acid sequences are provided in Supplementary Table S1.

### Modeling and docking of the DgcE-antibody complex

Based on the DgcE gene sequence and antibody variable region sequencing data, protein structures were predicted using AlphaFold3. Protein–protein docking was then performed with the HDOCK server (hdock.phys.hust.edu.cn) [16]. The resulting three-dimensional structures and docking poses were visualized using PyMOL. Residue-level interactions within the docked complex were analyzed using the DIMPLOT function in LigPlot^+ v.2.2^, and corresponding two-dimensional interaction diagrams were generated.

### Synergy assays

The synergistic activity between E11G12 and aminoglycosides against the parental APEC strain DE17 was evaluated using broth microdilution checkerboard assays (CLSI M07-A11). Bacterial suspensions were standardized to 5 × 10^5^ CFU/mL in cation-adjusted Mueller-Hinton broth (CAMHB), with 50 mg/L Ca^2+^ supplemented for aminoglycoside testing. Antibiotic panels included gentamicin (0.25–128 µg/mL; China Institute of Veterinary Drug Control), ciprofloxacin (0.03–16 µg/mL; fluoroquinolone, Sigma, 17850) and ceftazidime (0.06–32 µg/mL; β-lactam, Sigma SML0095). For the monoclonal antibody E11G12, the MIC was determined as the lowest concentration that resulted in no visible bacterial growth after 18 h of incubation at 37 °C. Since E11G12 alone exhibited no detectable inhibitory activity at concentrations up to 64 µg/mL, its MIC was defined as > 64 µg/mL for the purpose of FICI calculation. Consequently, the fractional inhibitory concentration index (FICI) was calculated as FICI = MIC (antibiotic+E11G12) / MIC (antibiotic) + MIC (E11G12 + antibiotic) / MIC (E11G12), with the denominator MIC (E11G12) set to the highest concentration tested (64 µg/mL) to avoid underestimation of synergy. Synergy was defined as FICI ≤ 0.5, additivity as 0.5 < FICI ≤ 1, indifference as 1 < FICI ≤ 4, and antagonism as FICI > 4. All experiments included *E. coli* ATCC 25,922 as a quality control strain and were performed in triplicate.

### In vitro time-kill curve assay

The synergistic activities of gentamicin (GEN), ceftazidime (CAZ), and E11G12 against APEC strain DE17 were evaluated using time-kill curves according to Reference [12]. Bacterial suspensions (5 × 10⁵ CFU/mL) were divided into six treatment groups: (a) Untreated control, (b) 1/2×MIC GEN (monotherapy), (c) 1/2×MIC CAZ (monotherapy), (d) 1/2×MIC E11G12 (monotherapy), (e) 1/2×MIC GEN + 1/2×MIC E11G12 (dual combination), (f) 1/2×MIC CAZ + 1/2×MIC E11G12 (dual combination). All tubes were incubated at 37 °C with shaking (120 rpm) for 24 h. Aliquots were collected at 0, 2, 4, 8, 12, and 24 h, and viable bacteria were quantified by serial dilution and plating.

### Statistical analysis

All data were analyzed using GraphPad Prism 9.0 with one-way ANOVA and Tukey’s post-hoc test; statistical significance was set at *p* < 0.05.

## Results

### Bioinformatic and structural characterization of DgcE

**Fig. 1:**
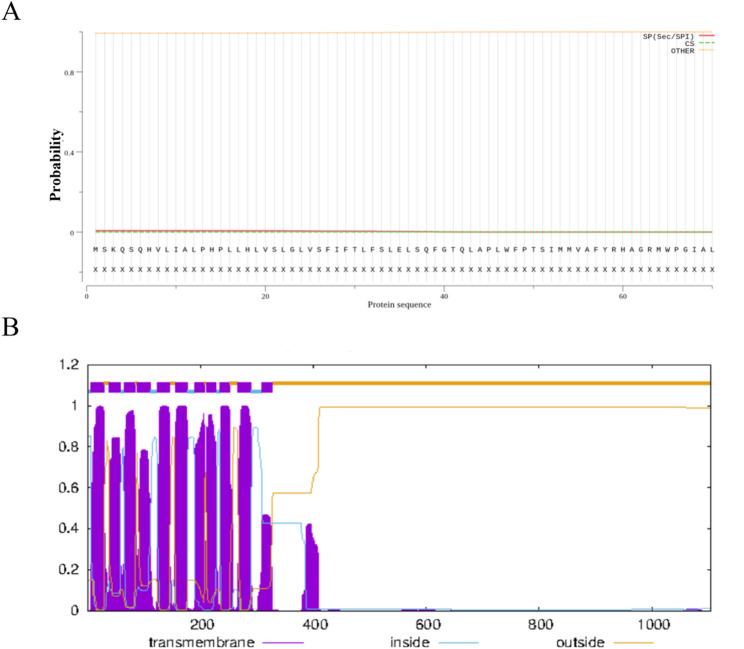
Structural Feature Analysis of DgcE. **A** Signal peptide prediction of DgcE ; **B** Transmembrane domain prediction of DgcE protein

Bioinformatic characterization identified DgcE as a 1,105-amino acid protein with a molecular weight of 123.95 kDa, a theoretical pI of 5.85, and the molecular formula C₅₅₉₅H₈₈₇₁N₁₄₉₅O₁₅₇₇S₅. Analysis with SignalP 5.0 confirmed the absence of a signal peptide (Fig. 1A). Furthermore, TMHMM 2.0 predicted 11 transmembrane domains (Fig. 1B), located at residues 7–29, 39–58, 65–84, 89–111, 124–146, 156–177, 190–207, 211–228, 235–252, 267–289, and 310–327, supporting a complex membrane-integrated topology.

### Functional validation of DgcE as a c-di-GMP synthase

**Fig. 2:**
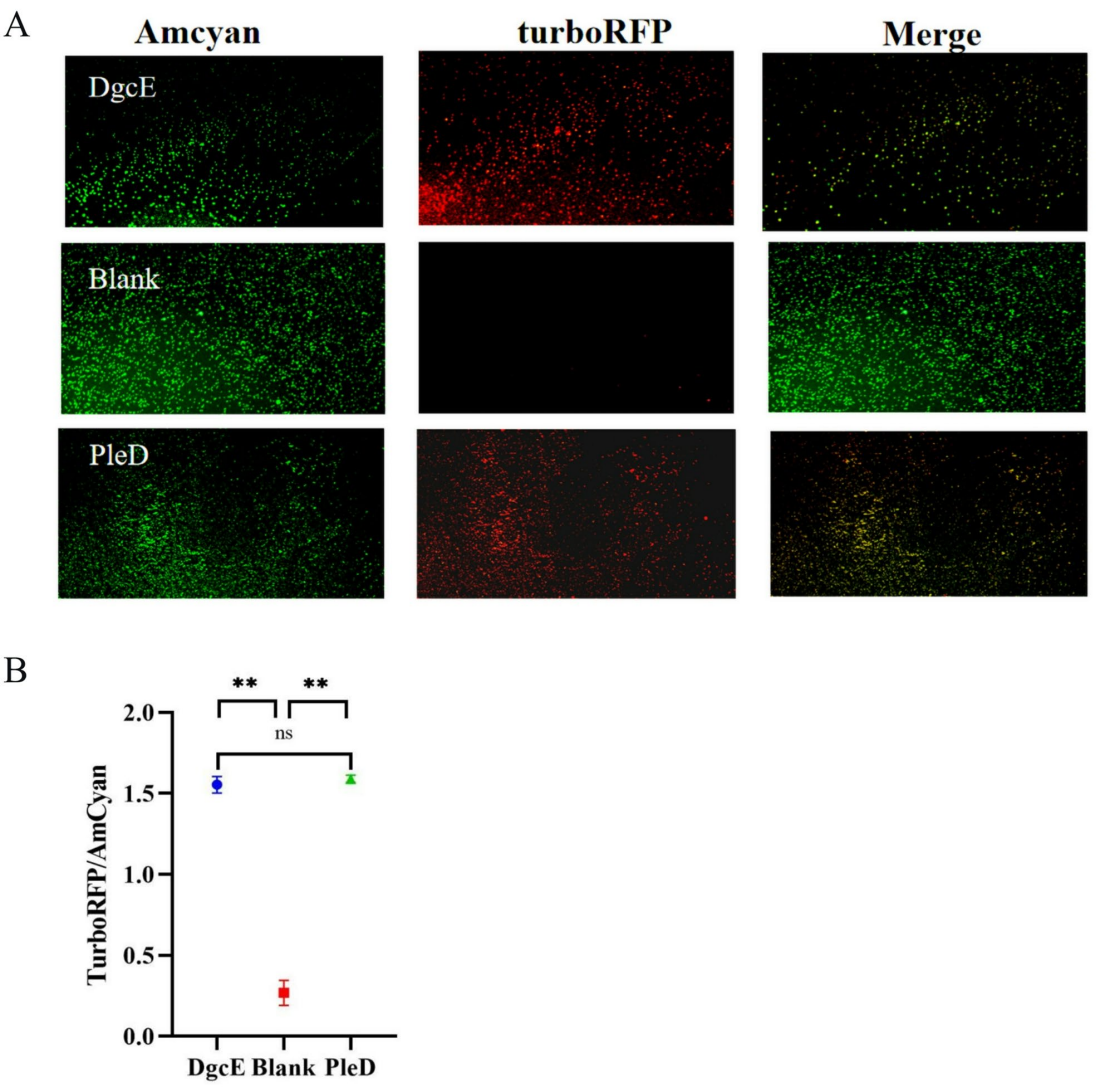
Functional identification of DgcE as a c-di-GMP synthase. **A** Dual-fluorescence reporter system with AmCyan and TurboRFP genes flanking a native triple-tandem riboswitch. Elevated c-di-GMP alters riboswitch conformation to activate downstream gene expression. PleD is a validated c-di-GMP synthase; **B** Quantitative analysis of c-di-GMP synthase activity. Each dataset consists of three independent biological replicates (*n* = 3), with error bars representing standard deviation (SD). Statistical significance was assessed using one-way ANOVA, with *P* < 0.05 considered significant (** for *P* < 0.01; ns indicates no significant difference, *P* ≥ 0.05)

To validate the c-di-GMP synthase activity of DgcE, we utilized a dual-fluorescence reporter system in which the expression of turboRFP was governed by three tandem c-di-GMP responsive riboswitches, while AmCyan served as a constitutive internal control. This design allows intracellular c-di-GMP accumulation to relieve riboswitch-mediated transcriptional repression, thereby inducing turboRFP expression. Following induction with 0.5 mM IPTG, both the DgcE and positive control (pleD) groups exhibited strong red fluorescence, whereas no detectable RFP signal was observed in the empty vector control (Blank) (Fig. 2A). Quantitative analysis revealed that the TurboRFP/AmCyan ratio was significantly elevated in the DgcE and PleD groups compared to the Blank control (*P* < 0.01), with the DgcE group showing a slightly higher ratio than the PleD group (Fig. 2B). These results conclusively demonstrate that DgcE functions as a diguanylate cyclase, providing a molecular foundation for further investigation into its regulatory mechanisms.

### DgcE protein purification and monoclonal antibody development

**Fig. 3:**
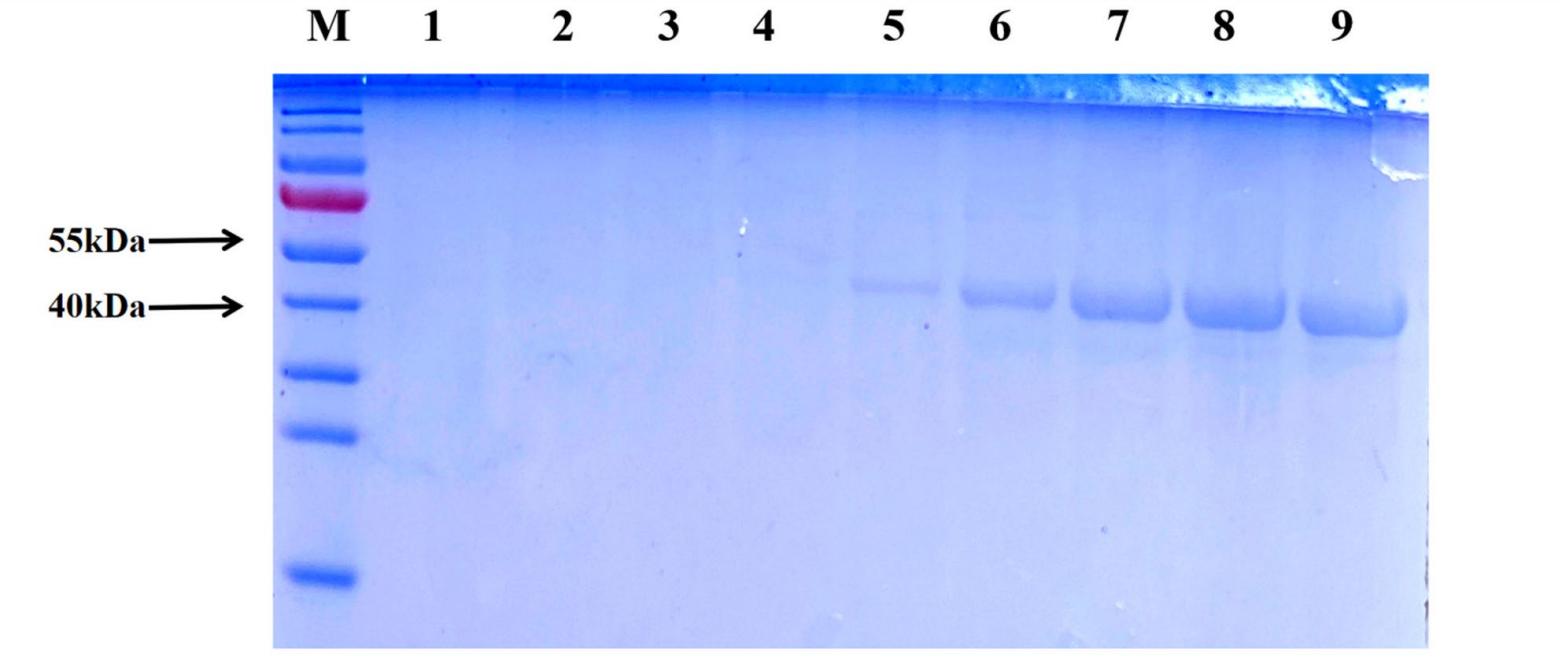
SDS-PAGE analysis of DgcE protein purification. M: Protein marker; Lane 1:Flow-through; Lanes 2–9:15mM, 20mM, 40mM, 60mM, 80mM, 100mM, 120mM, 150mM, 200mM imidazole

His-tagged DgcE was purified by nickel affinity chromatography. SDS-PAGE analysis indicated that the target protein (~ 124 kDa) reached optimal purity when eluted with 80–120 mM imidazole (Fig. 3, lanes 6–8). Eluates collected below 60 mM contained notable impurities (lanes 2–5), while imidazole concentrations exceeding 150 mM resulted in nonspecific co-elution (lane 9). After dialysis-based desalting and concentration, the final protein preparation was adjusted to 0.85 mg/mL as determined by BCA assay. Serum titers were determined by indirect ELISA with all samples tested in three technical replicates. The cut-off titer was defined as the highest dilution yielding an OD₄₅₀ value at least 2.1 times that of the negative control. As shown in Table 1, all immunized mice developed serum titers of 1:32,000. Mouse #1 showed the highest titer, with an OD₄₅₀ value of 0.817 ± 0.03 at a 1:32,000 dilution, significantly above the negative control (OD₄₅₀ = 0.055 ± 0.004), and was therefore selected for cell fusion and subsequent monoclonal antibody production.

**Table 1:** Results of ELISA detection of serum levels in mice after immunization

Dilution Factor Mouse ID	1 × 10^3^	2 × 10^3^	4 × 10^3^	8 × 10^3^	1.6 × 10^4^	3.2 × 10^4^	0
1	1.862 ± 0.04	1.720 ± 0.05	1.690 ± 0.07	1.425 ± 0.06	1.085 ± 0.04	0.817 ± 0.03	0.059 ± 0.005
2	1.788 ± 0.03	1.628 ± 0.06	1.593 ± 0.05	1.277 ± 0.04	0.957 ± 0.05	0.683 ± 0.03	0.060 ± 0.007
Negative Control	0.065 ± 0.006	0.068 ± 0.004	0.058 ± 0.005	0.061 ± 0.006	0.059 ± 0.005	0.055 ± 0.004	0.055 ± 0.005

### Production and functional characterization of E11G12 IgM-κ monoclonal antibody

**Fig. 4:**
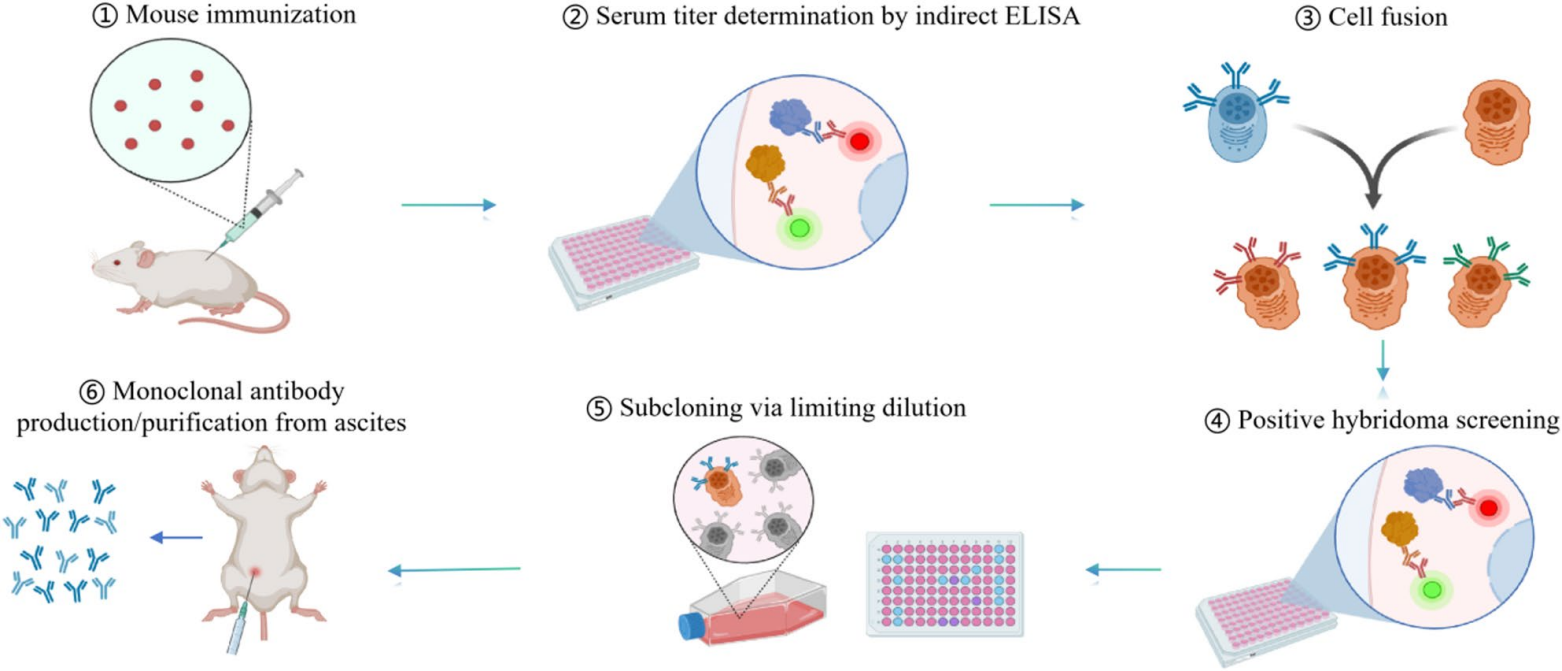
Flowchart of Monoclonal Antibody Production. The numbered panels (1–6) illustrate the schematic flowchart for monoclonal antibody production. Created with BioRender.com

The standardized preparation of the E11G12 monoclonal antibody comprised four sequential phases. The process commenced with antigen preparation, involving the purification of recombinant DgcE (124 kDa) via nickel affinity chromatography, which yielded endotoxin-free protein (< 0.1 EU/µg) at a concentration of 0.85 mg/mL following elution with 80–120 mM imidazole. For animal immunization, five BALB/c mice received four biweekly subcutaneous injections of 50 µg antigen emulsified in adjuvant. Mouse #1 exhibited the highest immune response, with a serum titer exceeding 1:32,000, and was selected for hybridoma generation. This phase involved the fusion of splenocytes from Mouse #1 with SP2/0 myeloma cells using PEG-1500, followed by HAT selection and three rounds of limiting dilution cloning, which successfully isolated the E11G12 clone. Finally, antibody production was achieved by expanding this clone in CD Hybridoma Medium, followed by concentration of the culture supernatant using a 50-kDa cutoff filter (Fig. 4). This integrated workflow enables the scalable production of functionally validated IgM-κ antibodies suitable for downstream conjugation.

Isotype analysis using a commercial ELISA kit confirmed that E11G12 belongs to the IgM-κ subclass. Quantitative assessment revealed robust reactivity with anti-IgM (OD₄₅₀ = 1.206 ± 0.08) and anti-κ (OD₄₅₀ = 1.351 ± 0.11) conjugates, while signals for other isotypes (IgA, IgG subclasses, and λ-chain) were negligible (OD₄₅₀ < 0.09), corresponding to a signal specificity of > 15-fold (Table 2). The pentameric conformation of IgM-κ antibodies promotes high-avidity binding to multimeric membrane proteins such as DgcE, supporting its potential for use in advanced therapeutic formats like antibody-antibiotic conjugates.

**Table 2:** Results of detection of E11G12 subtypes

Antibody Isotype	mAb ID
	E11G12
Goat Anti-Mouse IgA-HRP	0.087 ± 0.008
Goat Anti-Mouse IgG1, Human ads-HRP	0.067 ± 0.006
Goat Anti-Mouse IgG2a, Human ads-HRP	0.080 ± 0.009
Goat Anti-Mouse IgG2b, Human ads-HRP	0.073 ± 0.007
Goat Anti-Mouse IgG3, Human ads-HRP	0.069 ± 0.006
Goat Anti-Mouse IgM, Human ads-HRP	1.206 ± 0.08
Goat Anti-Mouse Kappa-HRP	1.351 ± 0.11
Goat Anti-Mouse Lambda-HRP	0.071 ± 0.007
Goat Anti-Mouse Ig, Human ads-HRP	0.069 ± 0.005
Goat Anti-Mouse Ig, Human ads-UNLB	0.076 ± 0.006

### Homology modeling and docking analysis of DgcE with a monoclonal antibody

**Fig. 5:**
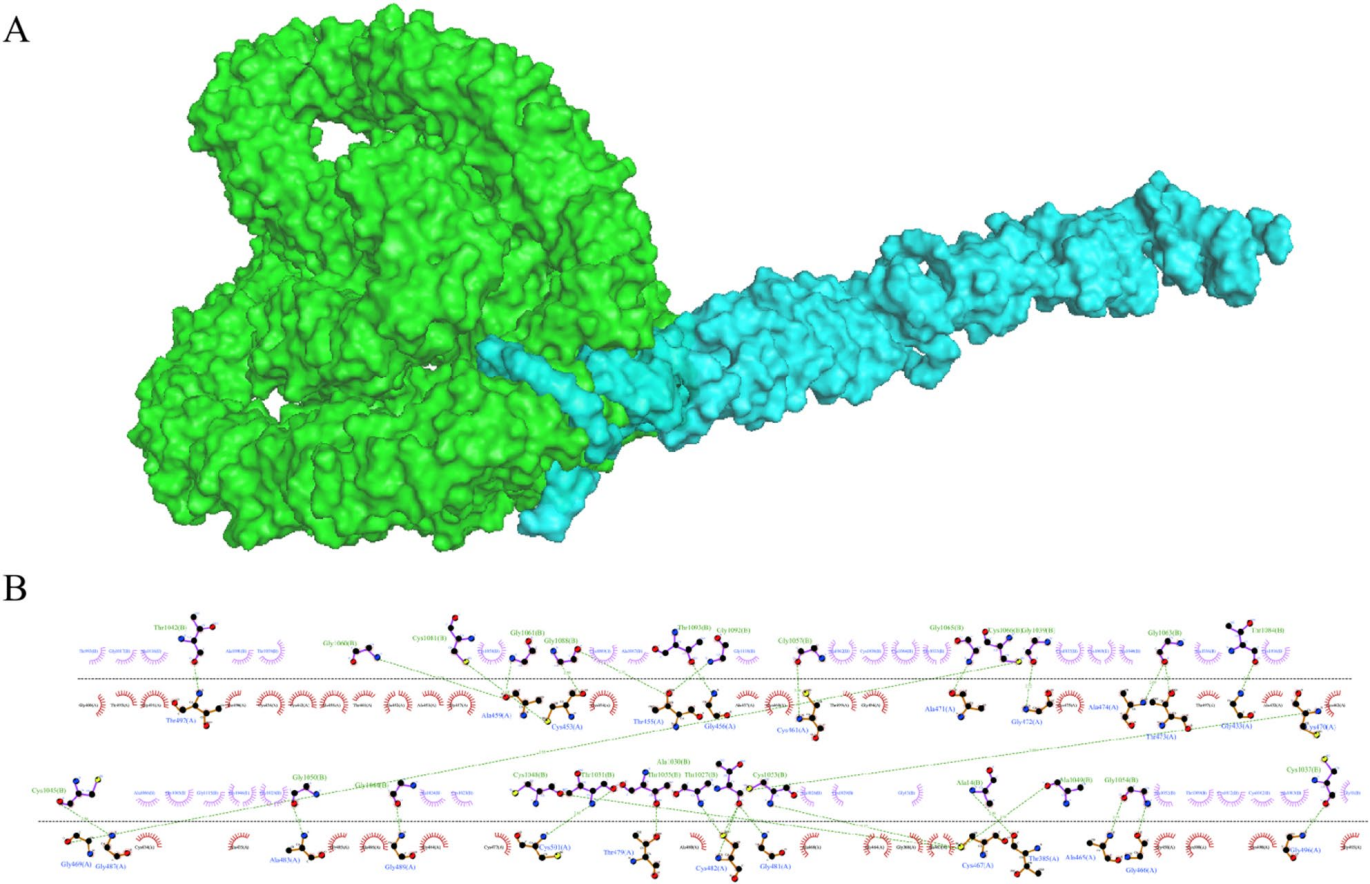
Protein Modeling and Docking Analysis of E11G12 with DgcE. **A** Protein–protein docking of DgcE and E11G12 with graphical representation of bound proteins. DgcE is shown in green and E11G12 is shown in blue. **B** Molecular interactions of docked complexes from LigPlot+ dimerization plot module. DgcE residues are above the dashed line and E11G12 residues are below. Green dashed lines indicate hydrogen bonds. Arcs show additional residues in the DgcE–E11G12 interaction

The structural model of E11G12 used for docking was generated based on the variable region sequences provided in Supplementary Table S1. To visualize the molecular interaction between DgcE and E11G12, we generated a homology model of DgcE and performed protein–protein docking using AlphaFold (Fig. 5A). Intermolecular interaction analysis with DIMPLOT identified several key hydrogen bonds, notably between DgcE residues Gly1060 and Gly1061 and E11G12 residue Ala459, as well as between DgcE residues Thr1031, Cys1053, and Ala1049 and E11G12 residue Cys467 (Fig. 5B). These specific interactions reflect strong structural complementarity and are consistent with a high-affinity binding interface between DgcE and E11G12.

### In vitro synergistic effects of E11G12 with aminoglycosides

**Fig. 6:**
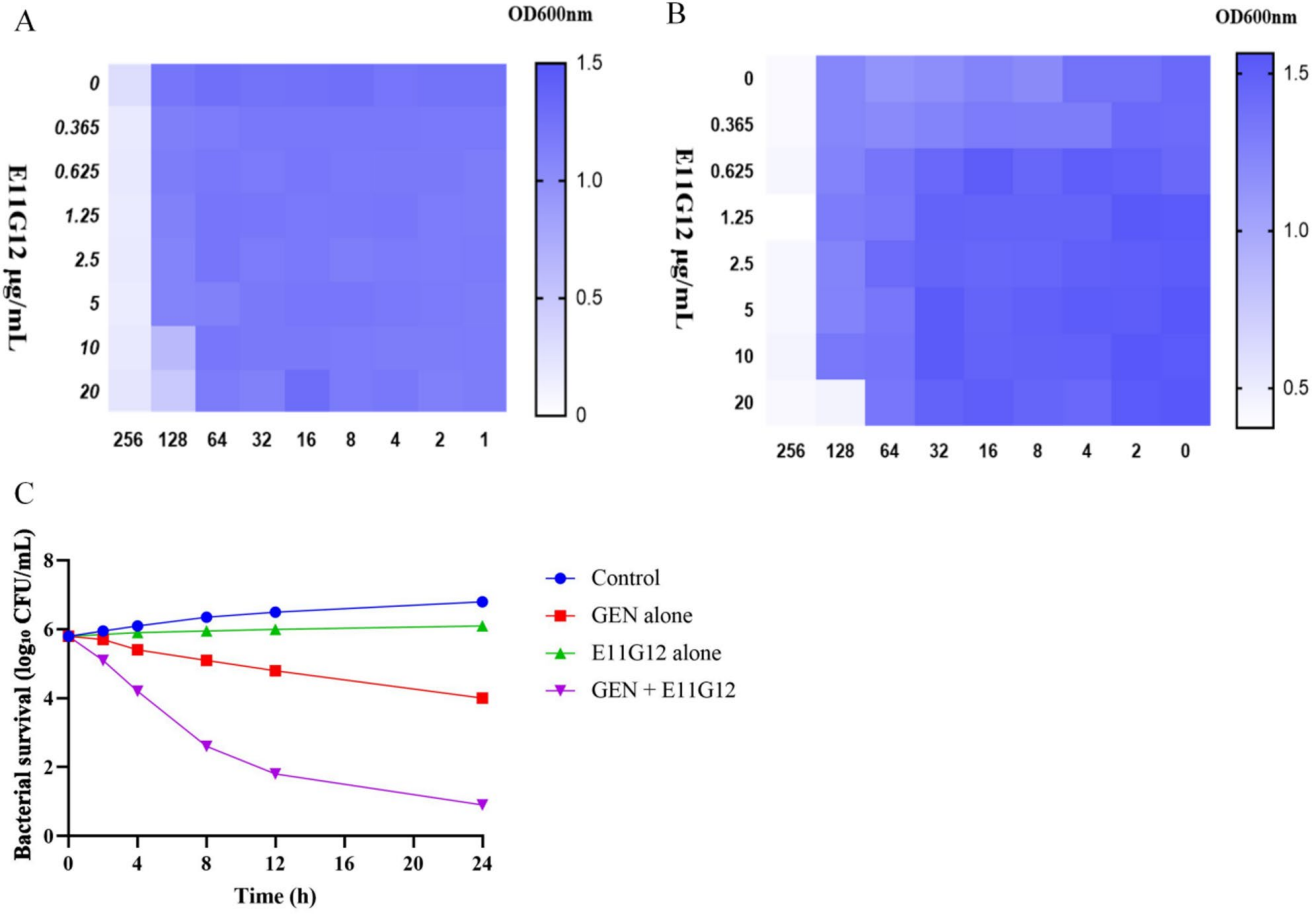
Results of the antibacterial experiment with E11G12 in combination with antibiotics. **A** Combined Antibacterial Activity with Gentamicin Sulfate; **B** Combined Antibacterial Activity with Ceftazidime; **C** Time-kill curve of E11G12 in combination with gentamicin against APEC DE17

Checkerboard assays were performed to evaluate the synergistic potential of E11G12 in combination with various antibiotics. E11G12 alone exhibited no detectable antibacterial activity at concentrations up to 64 µg/mL (MIC > 64 µg/mL). As shown in Table 3; Fig. 6A, E11G12 specifically enhanced the activity of gentamicin, reducing its MIC from 32 ± 2.1 µg/mL to 10 ± 1.8 µg/mL, with a fractional inhibitory concentration index (FICI) of 0.28 ± 0.05, indicating strong synergy (FICI ≤ 0.5). In contrast, only additive effects were observed when E11G12 was combined with ceftazidime (FICI = 0.92 ± 0.08) or ciprofloxacin (FICI = 0.95 ± 0.06) (Table 3; Fig. 6B). The synergistic interaction between E11G12 and gentamicin was further validated by time-kill kinetics (Fig. 6C). Treatment with gentamicin alone at 1/2×MIC (16 µg/mL) resulted in a modest reduction in bacterial counts, with regrowth observed after 8 h. E11G12 alone at 1/2×MIC (32 µg/mL) showed no significant bactericidal activity throughout the 24 h period. In contrast, the combination of gentamicin and E11G12 produced rapid and sustained bactericidal activity, reducing bacterial counts to 2.60 ± 0.15 log_10_ CFU/mL by 8 h and further to 0.90 ± 0.10 log_10_ CFU/mL by 24 h, representing a > 4 log_10_ reduction compared to the control. These results confirm the potent synergistic activity of E11G12 with gentamicin against APEC.

**Table 3:** Antibiotic synergy profiling with E11G12

Antibiotic	MIC Alone (µg/mL)	MIC with E11G12 (µg/mL)	FICI	Interpretation
Gentamicin	32 ± 2.1	10 ± 1.8	0.28± 0.05	Synergy
Ceftazidime	64 ± 3.7	58 ± 4.2	0.92± 0.08	Additive
Ciprofloxacin	8 ± 0.9	7 ± 1.1	0.95± 0.06	Additive

## Discussion

APEC is the causative agent of avian colibacillosis, a major bacterial disease affecting poultry across all age groups [17]. Clinical manifestations range from omphalitis in broilers to septicemia in both young and adult birds, often accompanied by systemic lesions such as perihepatitis and pericarditis [18, 19]. The considerable serotypic diversity and widespread distribution of APEC complicate its control [20]. This pathotype employs multiple virulence factors to establish extraintestinal infections, resulting in substantial economic losses in commercial poultry production and impairing animal welfare [21]. Antibiotics remain the primary strategy for managing APEC infections; however, their efficacy is increasingly compromised by the emergence of resistant strains, which present a growing threat to both animal and public health [20]. Alarmingly, APEC has developed resistance to nearly all antibiotic classes, a situation compounded by the extensive misuse of antimicrobials in poultry farming. Consequently, there is an urgent need to develop novel therapeutic agents or alternative strategies to combat APEC resistance and mitigate its impact on food safety and public health [22, 23].

Over recent years, monoclonal antibodies have emerged as promising alternatives for combating multidrug-resistant bacterial infections, owing to their high specificity, low propensity for inducing resistance, and demonstrated synergy with conventional antibiotics [24]. For instance, monoclonal antibodies targeting *Staphylococcus aureus* virulence factors, such as pore-forming toxins, have been shown to enhance the efficacy of vancomycin or linezolid in pneumonia models, improving survival by mitigating inflammatory damage and lung pathology [25, 26]. Despite these advances, the application of monoclonal antibodies in combination with antibiotics for treating APEC infections remains largely unexplored. To address this gap, the present study focused on DgcE—a key virulence-associated diguanylate cyclase in APEC—and generated an anti-DgcE IgM monoclonal antibody (E11G12) using hybridoma technology. E11G12 was designed to target the extracellular loops of the 11-transmembrane domain protein and was evaluated in combination with gentamicin. Peptide array epitope mapping and molecular docking analyses characterized antibody–antigen interactions, while checkerboard assays and FICI calculations assessed synergy with aminoglycosides. The results revealed that E11G12 reduced the gentamicin MIC by 68%, demonstrating specific and potent synergistic activity against APEC.

The ubiquitous bacterial second messenger c-di-GMP facilitates biofilm formation by performing diverse roles within complex regulatory networks. This signaling molecule is highly conserved across most bacteria [27] and governs a wide array of cellular phenotypes [28, 29]. Its synthesis is catalyzed by diguanylate cyclases (DGCs), which contain a C-terminal GGDEF domain responsible for dimerizing two GTP molecules. Mutations within the GGDEF catalytic motif can abolish c-di-GMP production [30, 31]. In *Escherichia coli*, DgcE—one of 12 DGCs—has been identified as a key regulator of extracellular matrix production during macrocolony biofilm development [32]. The ability of bacteria to adhere to host cells enables them to colonize and invade tissues, thereby facilitating biofilm formation and the establishment of infections [33]. Bacterial adhesion is recognized as the initial stage preceding biofilm development and represents a critical step in pathogenesis [34]. In the present study, the combination of E11G12 and gentamicin led to an 82% decrease in intracellular c-di-GMP levels and a 3.5-fold enhancement in gentamicin uptake. Collectively, these results demonstrate that E11G12 and gentamicin act synergistically, likely through antibody-mediated targeting of DgcE and subsequent disruption of c-di-GMP signaling, ultimately impairing bacterial adhesion, host-cell invasion, and biofilm formation.

A few points need to be kept in mind. One is that we only tested one APEC strain (DE17) in vitro. Whether the synergy with gentamicin holds for other serotypes or clinical isolates is still an open question. Another is that E11G12 is an IgM. While the pentameric structure gives good avidity, IgMs generally suffer from lower stability, shorter serum half-life, and greater manufacturing challenges compared to IgGs, and we did not examine these properties in vivo. Also, the absence of an animal infection model means we have no direct evidence on the efficacy or safety of the combination in a living host—including possible immunogenicity or off-target effects. Lastly, our proposed allosteric mechanism rests largely on docking and phenotypic correlations; direct structural data (co-crystallization or cryo-EM) are needed to confirm it. Future work should therefore include a broader range of APEC isolates, consider antibody engineering, and validate the combination in avian infection models.

## Conclusions

In conclusion, our findings demonstrate that the anti-DgcE monoclonal antibody E11G12, when combined with aminoglycosides, exhibits potent synergistic activity against APEC. This combination effectively counteracts antibiotic resistance through dual mechanisms—suppressing c-di-GMP-mediated virulence and enhancing drug uptake—thereby offering a promising therapeutic strategy for the control of drug-resistant APEC infections.

## Supplementary Information

Supplementary Material 1.

Supplementary Material 2.

Supplementary Material 3.

Supplementary Material 4.

Supplementary Material 5.

## Data Availability

The data used to support the findings of this study can be made available by the corresponding authors upon request.
