# Supplementary material for: An IgM monoclonal antibody targeting diguanylate cyclase DgcE potentiates gentamicin activity against avian pathogenic Escherichia coli through modulation of c-di-GMP signaling
Source: BMC Vet Res. 2026 Jun 1;22:455. doi: 10.1186/s12917-026-05548-y (PMC13440229; doi:10.1186/s12917-026-05548-y)
Supplement: Supplementary file 2 [file 12917_2026_5548_MOESM1_ESM.docx]

****Supplementary Table S1. Deduced amino acid sequences of the E11G12 monoclonal antibody variable regions.****

| **Antibody Chain** | **Amino acids Sequence** |
| --- | --- |
| ****Heavy Chain Variable Region (VH)**** | EVQLQQSGPELVKPGASVKISCKASGYSFTGYFMNWVKQSHGKSLEWIGRINPYNGATSYNQNFKDKATLTVDKSSSTAYMELRSLTSEDSAVYYCAREGGFAYWGQGTLVTVSA |
| ****Light Chain Variable Region (VL)**** | DIVMTQSPSSLTVTAGEKVTMSCKSSQSLLNSGNQKNYLTWYQQKPGQPPKLLIYWASTRESGVPDRFTGSGSGTDFTLTISSVQAEDLAVYYCQNDYFYPFTFGSGTKLEIK |
