# Supplementary material for: An IgM monoclonal antibody targeting diguanylate cyclase DgcE potentiates gentamicin activity against avian pathogenic Escherichia coli through modulation of c-di-GMP signaling
Source: BMC Vet Res. 2026 Jun 1;22:455. doi: 10.1186/s12917-026-05548-y (PMC13440229; doi:10.1186/s12917-026-05548-y)
Supplement: Supplementary file 3 [file 12917_2026_5548_MOESM4_ESM.docx]

****Details of Animal Euthanasia****

****1. Ethical Approval****
The animal study was approved by the Animal Care and Use Committee at the Shanghai Veterinary Research Institute, Chinese Academy of Agricultural Sciences (CAAS). All procedures involving animals were carried out in accordance with the guidelines established by the Institutional Administrative Committee (SYLAC/AM-M-L4043). Efforts were made throughout the study to minimize animal suffering.

****2. Animal Housing and Experimental Procedures****
Five female six‑week‑old specific‑pathogen‑free BALB/c mice (weighing 18–22 g) were housed under controlled environmental conditions (12 h light/dark cycle, 22 ± 2 °C, 50 ± 10 % humidity) with free access to sterilized food and water. Mice were housed in groups of five per cage. All procedures (immunization, blood collection, and euthanasia) were conducted during the light phase.

****3. Euthanasia Criteria and Procedures****

****3.1 Humane Endpoints****
Mice were monitored daily for signs of pain or distress (e.g., hunched posture, ruffled fur, lethargy, weight loss). Any animal meeting any of the following criteria would have been immediately euthanized, but none were reached during the study:

Severe lethargy or inability to right themselves within 15 seconds

Labored breathing or signs of severe respiratory distress

Persistent hunched posture with ruffled fur accompanied by weight loss exceeding 20 %

Moribund condition (unresponsive to gentle stimulation)

****3.2 Euthanasia Procedure****
Euthanasia was performed using isoflurane anesthesia followed by cervical dislocation. Mice were first placed in an induction chamber with 3-5% isoflurane delivered at 1 L/min oxygen flow until loss of consciousness was confirmed by the absence of the pedal reflex. Once deep anesthesia was confirmed, cervical dislocation was performed to ensure rapid and humane death. Death was verified by the cessation of respiration and heartbeat.

****3.3 Tissue Collection After Euthanasia****
No tissues were collected for experimental purposes because the mice were used only for monoclonal antibody production (splenocytes were collected before euthanasia for hybridoma fusion). After euthanasia, carcasses were disposed of according to institutional guidelines.

****4. Compliance with ARRIVE Guidelines****
This study adhered to the ARRIVE 2.0 guidelines. All animals were included in the final analysis (serum titers). No unexpected deaths occurred. The sample size (n = 5) was based on standard hybridoma protocols and was not determined by a priori power calculation.

****5. Statement****
All procedures involving animals were conducted in compliance with relevant regulations and institutional guidelines to minimize pain and distress. The study design and animal numbers were justified to ensure scientific validity while reducing overall animal use.
